# Robust magnetic polaron percolation in the antiferromagnetic CMR system EuCd_2_P_2_

**DOI:** 10.1038/s41535-026-00859-7

**Published:** 2026-02-04

**Authors:** Marvin Kopp, Charu Garg, Sarah Krebber, Kristin Kliemt, Cornelius Krellner, Sudhaman R. Balguri, Mira Mahendru, Fazel Tafti, Theodore L. Breeze, Nathan P. Bentley, Francis L. Pratt, Thomas J. Hicken, Hubertus Luetkens, Jonas A. Krieger, Stephen J. Blundell, Tom Lancaster, M. Victoria Ale Crivillero, Steffen Wirth, Jens Müller

**Affiliations:** 1 Institute of Physics, Goethe-University Frankfurt, Frankfurt (Main), Germany; 2 Department of Physics, Boston College, Chestnut Hill, MA USA; 3 Department of Physics, Durham University, Durham, UK; 4 ISIS Facility, STFC-Rutherford Appleton Laboratory, Harwell Science and Innovation Campus, Didcot, UK; 5 PSI Center for Neutron and Muon Sciences, Villigen PSI, Switzerland; 6 Department of Physics, Clarendon Laboratory, Oxford University, Oxford, UK; 7 Max-Planck-Institute for Chemical Physics of Solids, Dresden, Germany

**Keywords:** Electronic properties and materials, Magnetic properties and materials, Phase transitions and critical phenomena, Spintronics

## Abstract

The interplay between magnetism and charge transport is central to understanding colossal magnetoresistance (CMR), a phenomenon well studied in ferromagnets. Recently, antiferromagnetic (AFM) EuCd_2_P_2_ has attracted considerable interest due to its remarkable CMR, for which magnetic fluctuations and the formation of ferromagnetic clusters have been proposed as key mechanisms. Here we provide direct evidence that these effects originate from the formation and percolation of magnetic polarons. We employ a complementary set of sensitive probes that allows for a direct comparison of electronic and magnetic properties on multiple time scales revealing pronounced electronic and magnetic phase separation below T^*^ ≈ 2*T*_*N*_. These measurements indicate an inhomogeneous, percolating electronic system below T^*^ and well above the magnetic ordering temperature *T*_*N*_ = 11 K. In applied magnetic fields, the onset of the pronounced negative MR in the paramagnetic regime emerges at a universal critical magnetization. The characteristic size of the magnetic polarons near the percolation threshold is estimated to be ~6−10 nm. Our results establish dynamic polaron percolation within an AFM matrix as the microscopic origin of CMR in EuCd_2_P_2_, providing a unified framework for magnetotransport in Eu-based correlated semiconductors.

## Introduction

The observation that the magnetic state of a system critically affects its electronic transport properties is at the heart of spintronics research and applications^1^. One fundamentally important effect is the so-called colossal magnetoresistance (CMR), where the conductivity of materials drastically increases in a magnetic field, rendering such systems promising candidates for memory or sensor applications^2^. A wide variety of different material classes showing MR ratios ranging over of several orders of magnitude have been studied, among them europium chalcogenides, monoxide and hexaboride^3–8^, rare-earth perovskite manganites^9–16^, chromium spinels^17,18^, or pyrochlores^19,20^. These are often complex materials with competing magnetic, elastic, or orbital interactions leading to rich phase diagrams which may exhibit intrinsic (nonchemical) electronic phase separation resulting in transitions that are percolative in nature. In fact, nanoscale electronic phase separation is suggested to play a critical role not only for the CMR effect^15,21^, but also for high temperature superconductivity^22^ and in suppressing critical dynamics in quantum phase transitions^23^. Electronic phase separation, therefore, has been a subject of intensive recent theoretical and experimental interest, and magnetic field-dependent spatial inhomogeneities in the conductance of materials are thought to play a vital role in the magnetotransport effects in general^24^.

One particularly intriguing type of electronic inhomogeneity is the “large magnetic polaron”, first experimentally discussed for the case of a diluted magnetic semiconductor^25^ and theoretically described in refs. ^26,27^. Such a polaron describes a magnetically ordered quasiparticle consisting of a charge carrier trapped by strong exchange interaction to localized magnetic moments. Early indications for polaron formation were provided by magnetotransport measurements^3,28^. Formation and percolation of magnetic polarons have extensively been studied as underlying mechanism of the CMR effect in mixed-valence perovskite manganites^14,16^ and EuB_6_^7^. Methods like electron^29^ and scanning tunneling microscopy^30–32^ gave an approximate extension of the polarons of order a few nanometers. Further techniques comprise (small-angle) neutron scattering^33–35^, fluctuation spectroscopy^36,37^, and muon-spin relaxation (μSR)^38^.

The above-mentioned materials exhibit a ferromagnetic (FM) ground state. More recently, however, antiferromagnetic (AFM) materials have become of major interest for researchers as spintronic and quantum information technologies call for new materials without FM order^39–42^. Therefore, various AFM europium compounds, including Eu_5_In_2_Sb_6_^43,44^, Eu_5_In_2_As_6_^45^, and Eu*M*_2_*X*_2_ (*M* = Cd, Zn, Mn; *X* = As, P, Sb)^46–57^ have attracted attention, specifically those that show very large CMR effects. These materials provide a rich playground for possibly new quantum states^44^.

In this work, we focus on EuCd_2_P_2_, a trigonal compound with an A-type AFM ground state^51^. Recent reports have provided indications for polaron formation primarily based on magnetotransport^51,54,58,59^ and magneto-optic investigations^54^. Here, we present direct evidence for electronic phase separation by FM polaron formation and their percolation. In particular, we address the previously unexplored *dynamic* aspects of this phenomenon using resistance noise spectroscopy, weakly-nonlinear transport and μSR measurements at different time scales. Our findings are complemented by AC susceptibility, magnetotransport, and Hall effect measurements in order to provide a comprehensive picture of the correlations between magnetic and electronic properties. Beyond this, we aim to better understand the robustness of the polaron picture in view of the strong sensitivity of the electronic properties of EuCd_2_P_2_ to impurities and charge carrier doping^51,54,58,60^. This is particularly important since the material can be transformed from an AFM semiconductor exhibiting CMR to a ferromagnet with metallic behavior by changing the growth conditions^60^. We expect that our findings not only have ramifications for a deepened understanding of the CMR effect in general, but also help in providing an experimental base for better theoretical insight^61^.

## Results

### (Magneto)resistance

**Fig. 1:**
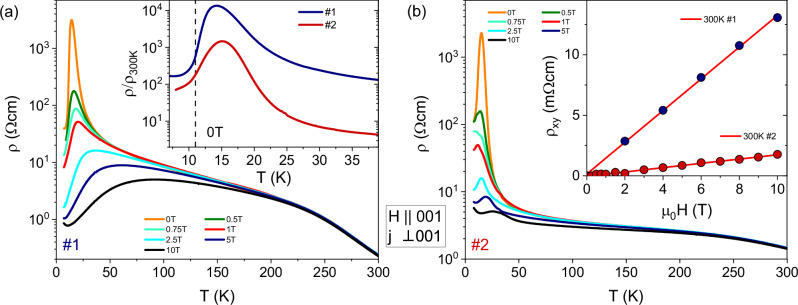
Electric characterization of two different EuCd_2_P_2_ single crystals. Comparison of the resistivities of sample #1 and sample #2 in (**a**, **b**), respectively, shown for *T* = 5−300 K in magnetic fields *μ*_0_*H* = 0−10 T aligned along the *c* axis. Inset in (**a**) shows the normalized zero-field resistivities for both samples at low temperatures and the dotted line indicates *T*_N_. Inset in (**b**) displays the measured Hall resistivities for both samples at *T* = 300 K. Lines are linear fits exhibiting a strong difference in slope (carrier density) for both samples in agreement with the differences in *ρ*(300 K).

Resistivity measurements along the basal *a*-*a* plane, with magnetic fields applied along the *c*-axis, were carried out using a standard four-terminal AC lock-in technique on two representative EuCd_2_P_2_ samples, one grown in the Frankfurt lab (sample #1) and one in the Boston lab (#2). The temperature-dependent resistivities for *T* = 5−300 K are shown in Fig. 1a, b in magnetic fields up to *μ*_0_*H* = 10 T for samples #1 and #2, respectively. In the inset of (a), the normalized zero-field resistivities *ρ*(*T*)/*ρ*(300 K) are shown for temperatures below *T* = 40 K. The overall qualitative behavior of *ρ*(*T*) of the two samples is similar, exhibiting a semiconducting temperature dependence (d*ρ*/d*T* < 0) upon cooling from 300 K down to a pronounced resistivity peak occurring at ${T}_{{\rm{peak}}}=T({\rho }_{\max })$, a few K above the AFM ordering temperature *T*_N_. For both samples we observe a distinct change of slope at about 240 K. However, there are a few remarkable quantitative differences. First, the room-temperature resistivity of sample #1 of *ρ*(300 K) = 0.23 *Ω* cm^59^ is significantly lower than the value of 1.4 *Ω* cm of sample #2. In comparison, the differences in the slopes of Hall resistivities measured at 300 K shown in the inset of Fig. 1b, which, in a simple one-band model, correspond to room-temperature carrier densities of *n*_*#*1_ = 4.8 × 10^17^ cm^−3^ and *n*_*#*2_ = 3.5 × 10^18^ cm^−3^. From this we can conclude that sample #1 shows a much larger mobility at room temperature μ(300 K) (see Table S1 in the SI). Importantly, both charge carrier concentrations are sufficiently low to allow the formation of magnetic polarons, which we shall discuss in more detail below.

Second, the relative increase in resistivity from room temperature to *T*_peak_ amounts to *ρ*(*T*_peak_)/*ρ*(300 K) = 1.33 × 10^4^ and 1.46 × 10^3^ for #1 and #2, respectively, while the peak temperatures are *T*_peak_ = 14 K and 15 K, in each case significantly above the AFM ordering temperature of *T*_N_ = (11.0 ± 0.2) K for both samples, as determined from magnetization measurements. It is important to note that the semiconducting behavior observed for the present samples for *T* > *T*_peak_ is markedly different from the metallic behavior (d*ρ*/d*T* > 0 for *T* > 75 K) for the sample described in refs. ^51,54^ with *T*_peak_ = 18 K and the same *T*_N_ = 11 K.

Third, both samples show a saturation of the resistivity or even an upturn, in particular in high magnetic fields, at the lowest measured temperatures.

Figure 1 also shows the resistivities of both samples in applied magnetic fields up to μ_0_*H* = 10 T along the *c*-axis, exhibiting the strongest suppression of more than four and three orders of magnitude for #1 and #2, respectively, for the highest applied field and at *T*_peak_. The quantitative differences between the samples become more obvious when plotting the usual magnetoresistance MR = [*ρ*(*B*) − *ρ*(0)]/*ρ*(0), see Fig. S2a in the Supplementary Information (SI). For a relatively large field of *μ*_0_*H* = 5 T, the maximum effect amounts to −99.95 % and −99.71 % for #1 and #2 at *T* = 14 K and 16 K, respectively. The MR sets in at higher temperatures for #1, where at μ_0_*H* = 5 T and a temperature of 50 K, it already exceeds −50 %. For a relatively small field μ_0_*H* = 0.1 T, the onset of the negative MR at *T* ~ 25−30 K is sharper and reaches values of about −40 % for both samples.

### Resistance fluctuation spectroscopy and weakly-nonlinear transport

**Fig. 2:**
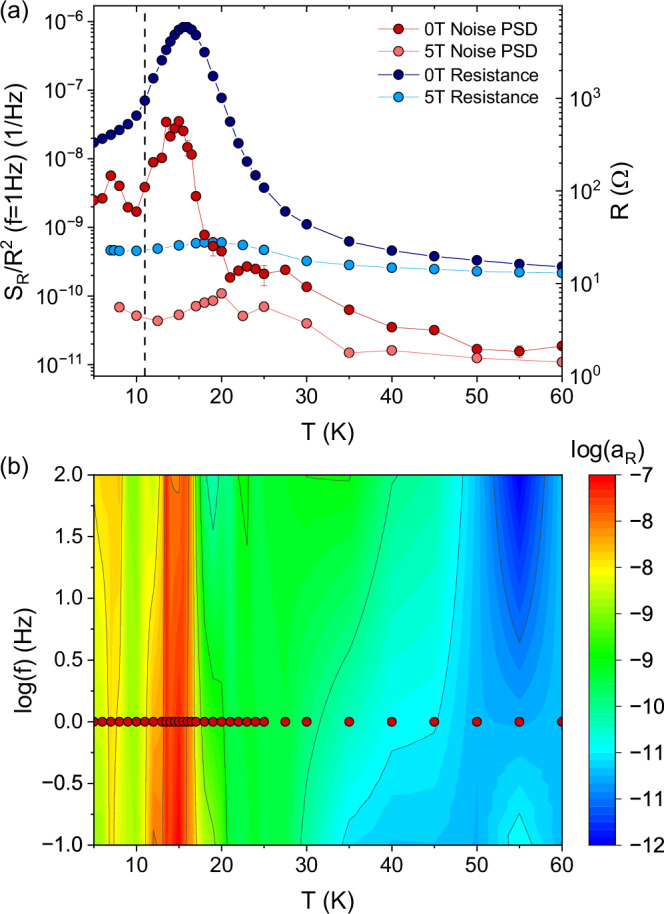
Low-frequency charge carrier dynamics of EuCd_2_P_2_. **a** Semilogarithmic plot of the normalized resistance noise power spectral density *S*_*R*_/*R*^2^(*T*, *f* = 1 Hz) (sample #2) (reddish colors, left axis) and the corresponding resistance *R*(*T*) (blueish colors, right axis). Dark red and dark blue denote the data in zero external field, the light colored data were measured in an external field of μ_0_*H* = 5 T. **b** Noise map showing the relative amplitude *a*_*R*_ = *f* × *S*_*R*_(*f*)/*R*^2^ in a logarithmic contour plot vs. temperature vs. $\log {\rm{frequency}}$ at zero magnetic field. The red dots correspond to the dark red data at *f* = 1 Hz shown in (**a**).

Measurements of the resistance noise power spectral density (PSD) *S*_*R*_(*f*, *T*) were carried out on sample #2 for temperatures *T* = 5−60 K. We discuss the generic 1/*f*^*α*^ noise with a frequency exponent *α* = 0.7−1.4, see “Methods”. In Fig. 2a, the noise magnitude *S*_*R*_/*R*^2^(*T*, *f* = 1 Hz) at zero magnetic field (dark red), together with the corresponding resistance *R*(*T*) (dark blue), are compared to their counterparts at μ_0_*H* = 5 T (noise: light red, resistance: light blue).

Upon cooling down from *T* = 60 K the noise level at *f* = 1 Hz slowly increases until about *T* = 30 K, where it reaches a plateau even though the resistance continues to strongly increase. Note that the plateau region of constant noise level becomes wider in temperature with increasing frequencies, see Fig. 2b, where the distribution of spectral weight is encoded in the color plot (on $\log$-scale) of the relative noise level *a*_*R*_ = *f* × *S*_*R*_(*f*)/*R*^2^, which is a dimensionless quantity characterizing the strength of the fluctuations, vs. temperature vs. $\log {\rm{frequency}}$. At temperatures below the plateau the resistance noise PSD then steeply increases by more than two orders of magnitude until it reaches a maximum at *T* ~ 13−15 K just below the resistance peak, before it drops to a minimum at about *T* = 10 K, the same temperature where the resistance changes slope (and shows an upturn in sample #1). Upon further cooling, a smaller peak at about *T* = 7 K is observed. In comparison to the fluctuations at zero magnetic field, the measured low-frequency noise in μ_0_*H* = 5 T weakly increases upon cooling from 60 K and becomes only weakly temperature dependent below about 20−25 K. The suppression of the normalized resistance noise in magnetic field sets in rather abruptly below these temperatures—the temperature region where the CMR is largest [see Fig. S2a]. Compared to zero field, the noise PSD for μ_0_*H* = 5 T is suppressed by −99.98 % at 15 K, which is essentially the same as for the MR. Thus, the mechanism responsible for the CMR is also responsible for the resistance fluctuations in this temperature regime.

**Fig. 3:**
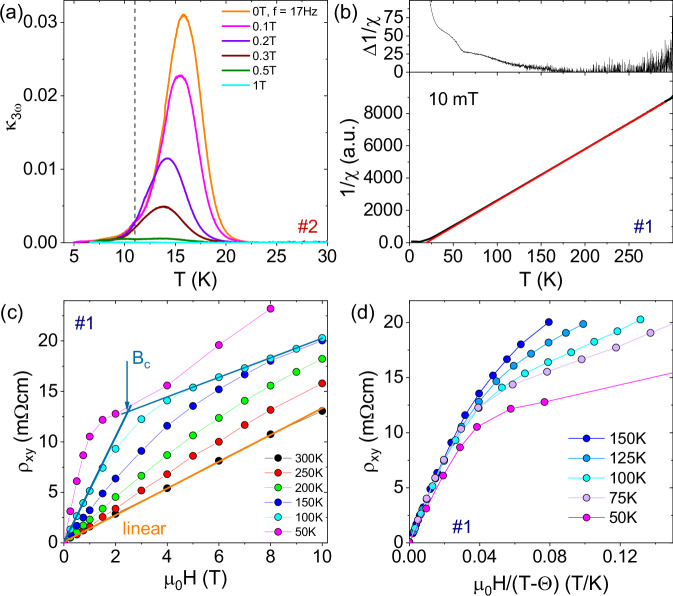
Transport and magnetic properties of EuCd_2_P_2_ single crystals. **a** Fourier coefficient *κ*_3*ω*_ = *V*_3*ω*_/*V*_1*ω*_ of the third-harmonic voltage vs. temperature in different magnetic fields for sample #2. **b** Inverse DC magnetic susceptibility 1/*χ*_dc_ measured at μ_0_*H* = 10 mT for sample #1 with a linear fit to the data at high temperatures in red extrapolating to *θ* = 20 K. The upper graph shows deviations of the data from this linear fit on the same temperature scale. **c** Hall resistivity *ρ*_*xy*_ measured at distinct temperatures for sample # 1. The curve gradually deviates from a purely linear behavior at 300 K (orange line) upon cooling. Blueish lines at *T* = 100 K represent linear slopes at small and large fields with a crossover field *B*_*c*_. **d** Hall resistivity *ρ*_*xy*_ for *T* = 150−50 K plotted versus the normalized field μ_0_*H*/(*T* − *θ*).

In basic percolation theory, often represented by the model of a simple random resistor network (RRN), weakly-nonlinear (third-harmonic) AC transport measurements probe microscopic inhomogeneities in the current distribution and are closely connected to the RRN’s 1/*f*-noise amplitude^62–64^ (see “Methods” part for details and SI for data of both samples). In Fig. 3a, the Fourier coefficient *κ*_3*ω*_ = *V*_3*ω*_/*V*_1*ω*_ of the third-harmonic transport measured at *f* = 17 Hz is shown for *T* = 5 − 30 K for sample #2 in different magnetic fields. In zero field, *κ*_3*ω*_ starts to increase strongly below about T^*^~22 K = 2 *T*_N_, coinciding with the increase of the resistance noise, and peaks at *T* = 15.5 K. This increase and peak is strongly suppressed already in small magnetic fields until at μ_0_*H* = 1 T *κ*_3*ω*_ ≈ 0, indicating a more uniform current distribution. The implications of this observation will be discussed below.

### Magnetic properties

The inverse magnetic DC susceptibility for sample #1 for a field of 10 mT displayed in Fig. 3b shows a Curie-Weiss behavior with an extrapolated paramagnetic Curie temperature of *θ* ≈ 20 K (bottom panel) and deviates from a linear behavior below about 150 K (top panel).

As shown in the inset of Fig. 1b, the room temperature Hall resistivity *ρ*_*xy*_(*B*) shows a linear behavior from which we have extracted the carrier concentrations for both samples in a simple one-band model. However, curvature in *ρ*_*xy*_(*B*) develops gradually upon cooling, until two distinct slopes at *μ*_0_*H* = 0 and at the maximum applied field of 10 T can be fitted to the data determining a crossover field *B*_*c*_ where the linear fits intersect, as exemplified for the data at 100 K in Fig. 3c. Such behavior is reminiscent of the anomalous contribution to the Hall effect in ferromagnets. Upon further decreasing the temperature, the slope change becomes more pronounced and the crossover field becomes smaller, until below *T* = 50 K the Hall resistivity exhibits a shoulder-like feature related to *B*_*c*_. A very similar behavior has been reported for EuCd_2_As_2_ where the anomalous contribution to the Hall effect was interpreted as the onset of quasi-static and quasi-long-range FM correlations^48^. For EuCd_2_P_2_, in the same temperature regime where a clear curvature in the Hall resistivity develops, the inverse magnetic susceptibility starts to deviate from linear behavior.

For the prototypical CMR system exhibiting magnetic polarons EuB_6_, Zhang et al.^65^ have demonstrated a nonlinear Hall effect as a signature of electronic phase separation. Following their arguments, we show in Fig. 3d the Hall resistivity *ρ*_*xy*_ vs. a normalized field *μ*_0_*H*/(*T* − *θ*). We find that for low fields the Hall resistivity curves collapse onto a single curve, indicating that the transition at *B*_*c*_ occurs at a single critical magnetization, very similar to the behavior of prototypical EuB_6_ and manganite CMR systems^65^. As shown in Fig. 5a below, the onset of the MR for each magnetic field coincides with the temperature of the corresponding switching field *B*_*c*_. This underscores the significant role of magnetic polaron percolation for the CMR effect in EuCd_2_P_2_.

**Fig. 4:**
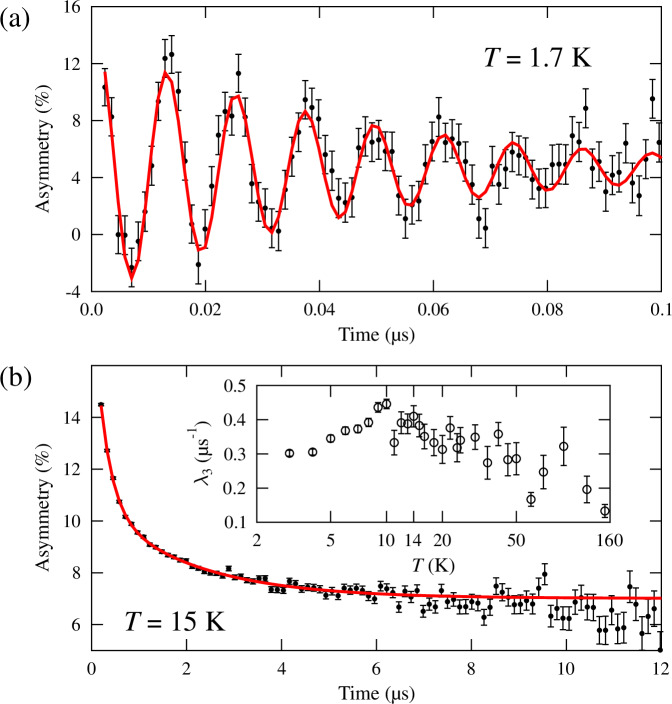
Muon-spin relaxation measurements on EuCd_2_P_2_ single crystals. Example spectra from ZF μSR measurements made at **a** SμS and **b** ISIS. Inset: low relaxation rate *λ*_3_ found in measurements made at ISIS.

In order to probe the local magnetic properties of the system and its dynamics we performed muon-spin relaxation (μSR) measurements on samples grown similarly to the method described in section Methods for sample #1. These measurements involve implanting spin-polarized muons in the material and measuring the subsequent muon-spin polarization, which is relaxed by the local magnetic field distribution at the muon sites. In zero-field (ZF) μSR measurements made at the Swiss Muon Source (SμS) we observe high-frequency oscillations in the μSR spectra for *T* < *T*_N_, see Fig. 4a, which result from muons experiencing a component of the local magnetic field transverse to the initial muon-spin direction. This behavior indicates that long-range magnetic order occurs in the sample at low *T*.

We fitted the early-time (*t*≤0.5*μ*s) part of the spectra measured below *T* = 11 K with a single oscillatory component using the function

1 $$A(t)={A}_{1}{{\rm{e}}}^{-{\lambda }_{1}t}\cos 2\pi \nu t+{A}_{{\rm{bg}}1},$$

where *A*_bg1_ accounts for muons with their initial spin direction parallel to the local magnetic field, or those muons that stop in the sample holder. We observe only 81 ± 2% of the total expected muon-spin polarization in this temperature region. This is made up of an oscillating part (accounting for 60 ± 6% of the polarization), corresponding to the precession of the component of the muon spin perpendicular to the local magnetic field, and a constant part (21 ± 7%), corresponding to the component of the muon spin pinned parallel to the local field (see SI for details). This implies the existence of another contribution to the measured asymmetry below *T*_N_ that is relaxed too quickly to be resolved in these measurements. We interpret this in terms of a phase separation between a magnetically-ordered majority phase of the sample (81% by volume), and a fluctuating minority phase (19%) whose muon response we cannot resolve.

**Fig. 5:**
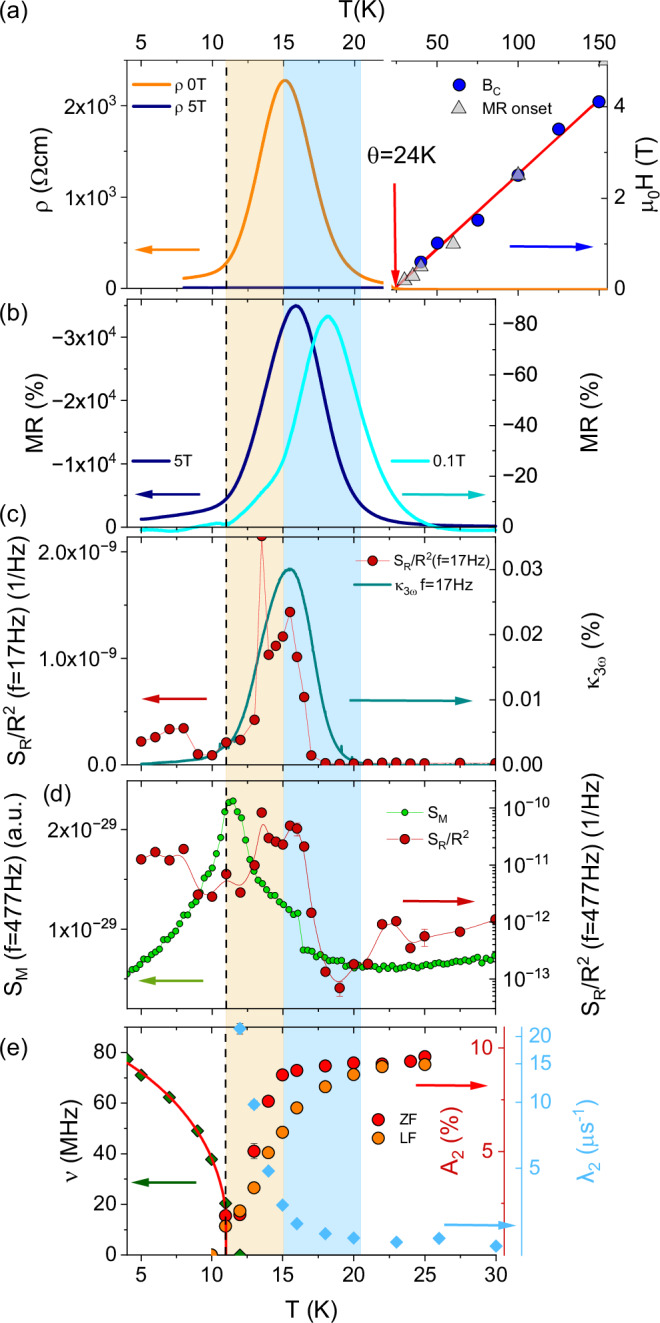
Compilation of different measurements on EuCd_2_P_2_. **a** Left: Resistance vs. temperature shown up to 2 *T*_N_ in zero-field (orange) and μ_0_*H* = 5 T (blue). Right: The crossover field *B*_*c*_ of the Hall resistivity (blue) and onset of MR (gray triangles) are shown up to *T* ~15 *T*_N_, with a linear fit to the data. **b** Magnetoresistance in high and small field of *μ*_0_*H* = 5 T and 0.1 T (left and right axis), respectively. **c** Normalized resistance noise PSD, *S*_*R*_/*R*^2^(*f* = 17 Hz) on a linear scale in comparison to the third-harmonic Fourier coefficient *κ*_3*ω*_. **d** Comparison of the calculated magnetic noise PSD *S*_*M*_(*f* = 477 Hz) (green) and the resistance noise PSD *S*_*R*_/*R*^2^(*f* = 477 Hz) (red). **e** (i) Oscillation frequency *ν* (green diamonds), (ii) relaxation rate *λ*_2_ (blue diamonds) and (iii) amplitude *A*_2_ in zero-field (dark red dots) and in longitudinal field (orange dots), measured by *μ*SR. The orange area highlights *T*_N_−*T*_peak_, the blue *T*_peak_−T^*^.

The fitted frequency *ν* = *γ*_μ_μ_0_*H*/2*π*, where μ_0_*H* is the magnitude of the local field at the muon site and *γ*_μ_ is the muon gyromagnetic ratio, follows an order parameter-like decrease with increasing temperature, see Fig. 5e. A fit to the functional form $\nu (T)=\nu (0){[1-{(T/{T}_{{\rm{N}}})}^{\alpha }]}^{\beta }$, see red line in Fig. 5e, yields *α* = 1.3(1) and *β* = 0.41(3), the latter being broadly consistent with fluctuations of Heisenberg moments in 3D close to the transition. We also estimate a transition temperature *T*_N_ = 11.2(1) K, and ground state frequency *ν*(*T* = 0) = 85.6 MHz, corresponding to a local field of 0.63 T.

Above *T*_N_, the spectra are found to relax exponentially, Fig. 4b, typical for depolarization due to dynamics in the local magnetic-field distribution in the fast-fluctuation limit^66^. The zero-field spectra can be modeled using a function $A(t)={A}_{2}{{\rm{e}}}^{-{\lambda }_{2}t}+{A}_{{\rm{bg2}}}$ with a relaxation rate *λ*_2_ that rapidly decreases in the region *T*_N_ < *T* ≤ T^*^ with a crossover temperature T^*^ ≈ 20−22 K. For *T* > T^*^, the relaxation rate *λ*_2_ remains at a roughly constant value around 2.5 μs^−1^, see Fig. 5e.

Complementary measurements were made at the ISIS muon source which are better suited to follow the evolution of the relaxation at high *T*. These reveal that the 2.5 μs^−1^ relaxation persists to *T* > 160 K. An additional slow relaxation, with rate *λ*_3_, is observed at ISIS and shown inset in Fig. 4b, that we discuss below. The limited time resolution at ISIS results in spectra that show a rapid change in relaxing amplitude *A*_2_ in the temperature region *T*_N_ < *T* ≲ 14 K (i.e., the *T* range where the relaxation rate changes most rapidly in data measured at SμS). These data provide another probe of this temperature regime, both in ZF and on the application of a longitudinal field (LF) of μ_0_*H* = 100 mT. The applied field is found to broaden this temperature regime, such that the temperature below which the amplitude starts to change increases to about T^*^. This broadening is reminiscent of the analogous effect seen in the resistivity peak on application of a field, Fig. 1a.

Finally, weak transverse field (wTF) measurements performed at S*μ*S are consistent with 30 ± 6 % of the volume of the sample being non-magnetic at *T* = 20 K (i.e., it does not relax the muon spins strongly). The remaining 70 ± 6% of the sample appears to host dynamically fluctuating magnetic moments that cause depolarization of the muon spins. Below *T*_N_, the measurements are consistent with the entire sample hosting magnetic moments although, as indicated above, 81% of the sample by volume hosts long-range magnetic order and the remaining 19% hosts fluctuations that cause rapid depolarization of the muons.

## Discussion

The main findings of the different methods are compiled in Fig. 5, where the magnetic transition is marked by the dashed line. Two important temperature regimes are marked by color: the orange area highlights the temperature range *T* = 11−15 K (between *T*_N_ and *T*_peak_) and the blue one up to *T* ~ 20−22 K(~T^*^ ≈ 2 *T*_N_). In (a) the resistance is shown on a linear scale in zero magnetic field (orange line) and μ_0_*H* = 5 T (blue) for temperatures up to 2 *T*_N_ (left). From *T* = 2 *T*_N_ up to *T* = 150 K (right), the crossover field *B*_*c*_ of the Hall resistivity is shown with a linear fit to the data, together with the onset of the MR (gray triangles). Despite the differences in carrier number of the present EuCd_2_P_2_ samples #1 and #2, their crossover fields *B*_*c*_ behave strikingly similar, showing essentially linear behavior below about 150 K extrapolating to *T*(*B*_*c*_ = 0) ≈ 24 K (roughly coinciding with the paramagnetic Curie temperature *θ*), below which weakly-nonlinear transport and negative MR set in, see also Fig. S5 in the SI.

For reasons of comparison with the literature^51,58,60^, in Fig. 5b, we present the magnetoresistance alternatively calculated by MR = [*ρ*(*B*) − *ρ*(*B* = 0 T)]/*ρ*(*B*) from the data in Fig. 1 for a large field of μ_0_*H* = 5 T (dark blue) on the left axis and a small field μ_0_*H* = 0.1 T (light blue) on the right axis. The onset of large MR roughly coincides with T^*^ ~ 2 *T*_N_ and its maxima are in the blue-colored regime *T*_peak_ < *T* < T^*^.

The temperature dependence of the normalized resistance noise PSD, *S*_*R*_/*R*^2^(*T*, *f* = 17 Hz), is shown in Fig. 5c on a linear scale (red circles) together with the third-harmonic resistance coefficient *κ*_3*ω*_ (blue line) for zero magnetic field. Obviously, there is a large increase in both, with the two effects roughly coinciding. A strong noise and third-harmonic resistance peak was also observed in perovskite manganites^36,63^ and in EuB_6_^37^, and is interpreted as a hallmark of a microscopically inhomogeneous current distribution caused by the percolation of magnetic polarons at the temperature-/magnetic field-induced insulator-metal transition in these FM CMR systems. Although the present system is considerably more complex than one-component random resistor networks (RRNs), which often serve as simple model systems in percolation theory^64,67,68^, this interpretation is based on the direct correlation between a strong increase in noise magnitude and a peak in weakly-nonlinear transport in a percolation scenario, i.e., when a conductive path through the sample is formed^62–64^. This is corroborated by the characteristic power-law scaling behavior of the scaled weakly-nonlinear transport signal *V*_3*ω*_/*I*^3^ vs. *R* with temperature as an implicit parameter, see Fig. S4 in the SI.

Figure 5d shows the comparison between 1/*f*-type magnetic noise PSD *S*_*M*_(*T*, *f* = 477 Hz) (linear scale, green circles) and resistance noise calculated for the same frequency (logarithmic scale, red) using the imaginary part of the AC magnetic susceptibility^68^:

2 $${S}_{M}(f)=V\frac{2{k}_{B}T}{\pi f}{\chi }^{{\prime\prime} }(f),$$

where *V* is the sample volume. In comparison to FM EuB_6_^37^, the peak in the resistance noise power spectral density for EuCd_2_P_2_ is not only larger in amplitude but also broader. Strikingly, the magnetic noise *S*_*M*_ obtained from measurements of the AC susceptibility is also of 1/*f*-type and shows a significant increase below *T* ≈ 100 K (see Fig. S6 in the SI). Upon cooling below 60 K, both types of fluctuations increase and exhibit a maximum at about 35 K, which means that the magnetic noise in this temperature regime is caused by equilibrium fluctuations of the magnetization, and that the resistance and magnetization are strongly coupled. Both *S*_*M*_ and the resistance noise PSD reveal a broad peak (local maximum) between *T* = 50 K and T^*^, see Fig. S6 in the SI. Upon further cooling, both these slow fluctuations strongly increase, whereas the peak in resistance noise coincides with the percolation threshold at *T*_peak_ and the peak in magnetic noise with *T*_N_. The magnetic fluctuations appear to be driven by a competition between FM and AFM correlations, until the system orders antiferromagnetically.

In Fig. 5e, the main results of the *μ*SR measurements, in part already discussed in section Magnetic properties above, are displayed. From μSR, the system shows magnetic order below *T*_N_ = 11 K. In the temperature regime *T*_N_ < *T* < T^*^ EuCd_2_P_2_ is not long-range magnetically ordered, but there are dynamic fluctuations in the local magnetic field that rapidly relax the muon-spin polarization. Assuming a Redfield model for relaxation in the fast-fluctuation regime, we expect ${\lambda }_{2}\propto {\gamma }_{\mu }^{2}{B}_{{\rm{a}}}^{2}\tau$, where *B*_a_ is the amplitude of the fluctuating field. The relaxation rate of 20 μs^−1^ seen just above *T*_N_ then corresponds to a fluctuation time of order *τ* ≈ 0.1 ns. The rapid collapse of the relaxation rate for *T*_N_ < *T* < T^*^ implies that the cause of the rapid relaxation vanishes above T^*^ and a distinct relaxation channel depolarizes the muons, with a characteristic fluctuation time an order of magnitude smaller. It is notable that the characteristic temperature T^*^ coincides with the onset of magnetic polaron percolation which implies that the same underlying electronic structure is responsible for both effects, despite their dependence on very different energy scales. This holds for both samples with very different charge carrier concentration indicating an intrinsic effect. In fact, a possible explanation of the muon response would be the occurrence of regions of slowly fluctuating magnetic moments locking together upon cooling below T^*^, providing the broad spectral density of fluctuations required to relax the muons. Simulations of the local field distribution (see Methods) suggest that an AFM model largely accounts for the muon response in the ordered regime. However, the measurements at ISIS feature evidence for an additional, slowly-relaxing background component, not present in the measurements made at SμS. The fitted relaxation rate, *λ*_3_, for this component is shown in the inset of Fig. 4b, where we can see features that correlate with *T*_N_ and T^*^ (increase below about 20 K). This contribution to the spectra likely results from muons stopping outside the sample in the silver backing plate (which is absent at S*μ*S) and might imply that the sample has a small FM response in its fluctuations, such that muons stopping outside the sample are relaxed parasitically. Importantly, the *μ*SR provides evidence for phase separation related to polaron formation. Just above *T*_N_, the strong relaxation from the majority phase of the sample (approximately 70% by volume) can be accounted for by muons stopping in magnetically fluctuating regions, with the remainder giving a non-magnetic response. Below *T*_N_, the fact that only 81% of the muon polarization is accounted for by muons experiencing long-range magnetic order is evidence for the occurrence of a long-range antiferromagnetically ordered majority phase and a minortity phase (approximately 19% by volume) that is magnetically fluctuating, i.e., where the muons feel an environment different to the conventional AFM order such as, e.g., domain walls or regions close to FM clusters. These fluctuations are likely to be dynamic on the muon timescale, but could also be the result of quasistatic disordered fields.

**Fig. 6:**
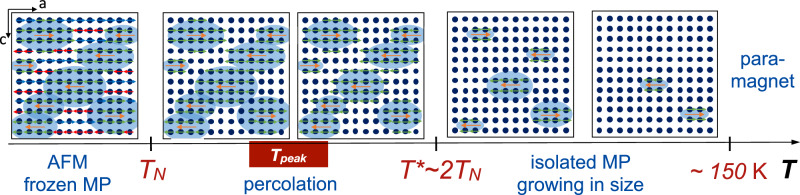
Magnetic polaron percolation model for EuCd_2_P_2_. Schematics of the suggested model of magnetic polaron (MP) percolation in EuCd_2_P_2_ in zero magnetic field. At T^*^ ~ 2*T*_*N*_, roughly coinciding with the paramagnetic Curie temperature *θ*, both MP percolation and large negative MR emerge at zero field at a universal critical magnetization which shifts linearly to higher temperatures with field, see text and Fig. 5a. *T*_peak_ marks the range of the percolation threshold (and largest CMR) in different samples. Connected FM clusters of presumably ellipsoidal shape remain frozen in the AFM ordered phase below *T*_*N*_ (dimensions not to scale).

Based on these findings, we arrive at a comprehensive picture schematically shown in Fig. 6. In order to clarify the origin of the resistivity peak at *T*_peak_ above *T*_*N*_ and its strong suppression in magnetic field (CMR effect) we propose the formation and percolation of nanoscale FM clusters, i.e., magnetic polarons, which start to dynamically form at elevated temperatures ~150 K, where first deviations from a Curie-Weiss law are observed in the magnetic susceptibility and the curvature in the Hall effect starts to develop as isolated clusters grow in size upon cooling. (We note that also around 50 K a stronger increase in Δ1/*χ* coincides with the 1/*f*-noise starting to significantly increase.) Sunko et al.^54^ were able to trace the signature of FM correlations up to T^*^ ≈ 2 *T*_N_. Indeed, our findings of a strongly enhanced 1/*f*-type resistance noise PSD and the onset of a pronounced weakly-nonlinear transport signal (third-harmonic voltage) directly reveals dynamic fluctuations of the connected FM clusters and the presence of an inhomogeneous electronic system below T^*^, in agreement with evidence for magnetic phase separation from *μ*SR. The percolation threshold is identified as *T*_peak_, where the magnetic polarons overlap and form a conducting path through the sample, which accounts for the strong decrease in resistivity. Our finding of magnetic phase separation of order ~10−20% agrees well with the critical volume fraction in different percolation scenarios in three-dimensional lattices^69^. In this picture, we interpret the nonlinear Hall effect as a signature of electronic phase separation^65^ describing both the onset of percolation and large negative MR in increasing magnetic fields at a universal critical magnetization. The extrapolation of the switching field *B*_*c*_ in the Hall effect yields T^*^ as the temperature where stable magnetic polarons start to percolate in zero external field. Finally, the amplitude and relaxation rate in *μ*SR measurements deviate from their high-temperature behavior below T^*^, indicating that the inhomogeneous system’s fluctuations also occur on very fast timescales. Below *T*_N_ the magnetic polarons freeze in the AFM matrix and continue to influence the magnetic and electronic properties of the system^70^.

In order to get an estimate of the size of the FM clusters, we consider the model of correlated polarons by Bhatt et al. and Kaminski and Das Sarma^71,72^. For temperatures smaller than a characteristic energy scale given by the localization length of a dopant (captured hole or electron), all magnetic spins within distance ${r}_{p}(T)=\frac{1}{2}{a}_{B}ln(sS| {J}_{0}| /{k}_{B}T)$ of dopants align, forming a magnetically polarized cluster (polaron). Here, *a*_*B*_ is the localization length of the donor, *s* the carrier spin, *S* the spin of the Eu^2+^ in the matrix, and *J*_0_ the effective exchange constant between the carrier and local moment spins. In ref. ^63^, it is suggested that the magnitude of the weakly-nonlinear transport signal *κ*_3*ω*_ in a manganite thin film is directly related to the concentration of correlated polarons. With our finding of *κ*_3*ω*_ ≈ 3% corresponding to the ratio of available carriers participating in magnetic polaron formation at *T*_peak_, and our estimated ‘infinite cluster’ FM volume of order 10−20%, we can roughly estimate the size of magnetic polarons at *T*_peak_. Assuming for simplicity randomly overlapping spherical bound magnetic polarons we find *r*_*p*_(*T*_peak_) ~ 6−10 nm for the two samples investigated here, a reasonable size of the critical percolation radius, which is also in line with the polaron size in EuB_6_^32^. We note, however, that in the electronically and magnetically anisotropic compound EuCd_2_P_2_ the magnetic polarons are not expected to be spherical but rather shaped like an ellipsoid with the long axis along the in-plane easy magnetization direction^61,70,73–75^, see the schematic Fig. 6.

Finally, we mention that a comparison of various parameters relevant for the magnetotransport properties for different samples with a significant range of charge carrier concentration *n*_*c*_ reported in the literature and in this work (see Table S1 in the SI) highlight the strong competition between FM and AFM interactions in this material (some samples are even reported to be FM metals down to low temperatures^60^). All samples that show a peak in the resistance due to a semiconducting behavior at low temperatures exhibit a CMR effect above the AFM ordering. Magnetic polaron formation, therefore, is a robust feature in EuCd_2_P_2_ and likely also in other members of this class of materials where the competition of AFM and FM interactions of localized spins and low charge carrier concentration gives rise to large magnetotransport effects.

## Methods

### Sample growth

Single crystals of EuCd_2_P_2_ have been succesfully grown from Sn flux, with two distinct samples resulting from slightly different growth processes provided by the institutions in Frankfurt (sample #1) and Boston (sample #2). The first sample (#1) was grown using ingots of europium (99.99%, Evochem), teardrops of cadmium (99.9999%, Chempur), red phosphorous (99.9999 %, Chempur) and tin (99.999%, Evochem) as starting materials. In the growth of sample #2 sublimed ingots of europium (Alfa Aesar, 99.9%), cadmium tear drops (Alfa Aesar, 99.95%), red amorphous phosphorus powder (Alfa Aesar, 98.9%), and tin shots (Alfa Aesar, 99.999%) were used.

The elements were then cut into pieces and mixed together with a stoichiometry of Eu:Cd:P:Sn = 1:2:2:20 under an inert Ar atmosphere inside a glove box. The materials were then placed in a crucible (#1: graphite, #2: alumina) inside an evacuated quartz ampule. In #1 the elements were heated to 450 °C and held at this temperature for 5 h. This ensured that the phosphorus gradually reacted with the other elements. Subsequently, the temperature was increased to 850 °C and held at this temperature for several hours in order to homogenize the melt. The temperature was then gradually decreased to 600 °C at a rate of 2 K/h, where the Sn flux was removed by centrifugation, resulting in samples with a size of 3 × 3 × 1 mm^3^. In the growth of the second sample (#2), a similar procedure was followed, with the exception that the temperature was increased to 950 °C and held for 36 h. Following this, the temperature was then reduced to 550 °C at a rate of 3 K/h, where the flux was then removed by centrifugation, resulting in samples of similar size. The main difference between the two crystal growth methods are the choice of the inner crucible, the purity of the elements, and the temperature profile during the growth, which all could influence the overall quality of the crystals, in particular the concentration of intrinsic doping.

### Transport measurements

Resistance measurements were carried out on two samples using a standard AC four-point technique with a lock-in amplifier (e.g., SR830). To ensure good ohmic contacts, the sample surfaces were coated with thermally-evaporated gold (200 nm) using a wetting layer of chromium (7 nm) in a desired contact geometry, and then contacted with gold wires and conducting silver paste. The current was applied in the *a*-*a* plane and magnetic fields always aligned to the *c*-axis. The third-harmonic resistance measurements were carried out in the same configuration. Care has been taken that the applied currents were sufficiently small to avoid sample heating. In order to rule out second-order effects of third-harmonic voltage generation caused by self-heating due to a strong temperature dependence of the resistivity at the percolation transition, a simulation using a simple heat conduction model has been performed. As expected, such a third-harmonic signal mimics d*R*/*dT*, i.e., shows negative values at the high-temperature flank of the resistivity peak, which is, however, *not* observed in our experiment. For the Hall measurements, the contact geometry with the smallest longitudinal component was used, and the voltages for discrete postive and negative field values have been antisymmetrized. Measurements of the noise power spectral density were carried out in a four-point configuration similar to a standard resistance measurement. Here the voltage measured from the sample is first amplified by a low noise amplifier (e.g., SR560) and the signal is then processed by a signal analyser (SR785) calculating the Fourier transformation and delivering the power spectral density

3 $${S}_{R}(f)=\mathop{{\mathrm{lim}}}\limits_{T\to \infty }\frac{1}{T}{\left|\mathop{\int }\limits^{T/2}_{-T/2}\delta R(t){{\rm{e}}}^{-2\pi ift}{\rm{d}}t\right|}^{2}\,.$$

A cross correlation with two voltage amplifiers and two lock-in amplifiers as described in ref. ^76^ was used to further reduce the noise background from the setup. For the present sample, at certain temperatures Lorentzian spectra superimposed on the underlying 1/*f*-type noise have been observed, a signature of two-level processes with finite lifetimes. By fitting the measured spectra at different temperatures with a superposition of 1/*f*-type and Lorentzian noise PSD (see SI Fig. S3), we obtain the magnitude and corner frequency of the Lorentzian and the magnitude *S*_*R*_/*R*^2^(*T*, *f* = 1 Hz) and frequency exponent *α*(*T*) of the underlying 1/*f*-type noise. Since the intermittently occurring two-level fluctuations showed no clear systematics in the measured temperature intervals, we focus in this work on the generic 1/*f*^*α*^ noise.

### Susceptibility

DC susceptibility measurements were conducted using the vibrating sample magnetometry option of the Physical Property Measurement System (PPMS) by Quantum design. Further magnetic measurements were conducted using a magnetic property measurement systems (MPMS3, Quantum Design Inc., San Diego, CA, USA). The magnetic AC susceptibility *χ*_AC_ was measured along the (001) axis (*c*-axis) with an applied AC field of 5 Oe after cooling in zero applied field (ZFC). We note that the magnetic properties of EuCd_2_P_2_ are highly sensitive to applied magnetic fields and therefore, the remnant field of the superconducting magnet was determined for each cool-down (typically 25 Oe) and compensated for.

The fluctuation-dissipation relations express the noise PSD in the equilibrium state of a system in terms of the dissipative part of the linear response of the same system. For comparison with the measured resistance noise, which comprises a sum of different contributions we calculated the PSD of the magnetic fluctuations, *S*_*M*_(*f*), using the imaginary part of the AC susceptibility after Eq. (2).

### μSR

A mosaic of single crystals of EuCd_2_P_2_ was mounted on a silver foil (foil thickness 25 μm) with the *c*-axis out of the plane of the mosaic and parallel to the incoming muon beam. We made zero-field (ZF) μSR measurements^66^ using the FLAME instrument at the SμS with the initial muon-spin direction at 45° to the *c*-axis in the temperature range 1.5–30 K using a ^4^He cryostat and ZF, LF, and wTF measurements at ISIS using the EMU spectrometer with the initial muon-spin direction parallel to the *c*-axis. For the ISIS measurements, the sample was mounted on an Ag plate inside a ^4^He cryostat. Fitting functions for the SμS data are described in the main text. For ISIS ZF data, we used a fitting function

4 $$A(t)={A}_{2}{{\rm{e}}}^{-{\lambda }_{2}t}+{A}_{3}{{\rm{e}}}^{-{\lambda }_{3}t}+{A}_{{\rm{bg}}3}.$$

We found that *A*_3_ could be held constant at two different values over these two regions.

We also completed supporting DFT calculations to find candidate muon stopping sites within the crystal using the MuFinder programme with the CASTEP code^77,78^. A 2 × 2 × 2 supercell was populated with a muon at a random position and the geometry was optimized to find a local minimum in energy, and this process was repeated 46 times. Calculations were done using the PBE functional with a 2 × 2 × 2 k-point grid using a cutoff of 550 eV. Muons were then moved to symmetry-equivalent positions and grouped based on position. A low-energy site was found for muons aligned along the *c* axis with the Cd and P ions, 0.5 Å above and below the *a*-*a* plane of the Eu ions. We are able to calculate a local dipole field distribution experienced by muons at this position, from which muon spectra may be simulated. The AFM order at low temperatures contains FM layers in the *a*-*a* plane, which are antiferromagnetically coupled along the *c* axis. From this ordering, we constructed spectra which we found to be in good agreement with our experimental data for an Eu ordered moment of 5.4μ_B_. Although this value is reduced compared to the full moment of the magnetic Eu ions (7μ_B_), our calculation does not account for the hyperfine field at the muon site, which could account for some of the discrepancy.

## Supplementary information

Supplementary information

## Data Availability

All data are available in the main text or the supplementary materials. Data taken in the Frankfurt and Dresden labs will be made available via 10.5281/zenodo.18450854. Data from the UK effort will be made available via 10.15128/r176537145s.
