# Supplementary material for: Robust magnetic polaron percolation in the antiferromagnetic CMR system EuCd2P2
Source: NPJ Quantum Mater. 2026 Feb 4;11(1):22. doi: 10.1038/s41535-026-00859-7 (PMC12979192; doi:10.1038/s41535-026-00859-7)
Supplement: Supplementary file 1 — Supplementary information [file 41535_2026_859_MOESM1_ESM.pdf]

Robust Magnetic Polaron Percolation in the Antiferromagnetic CMR System $EuCd_2P_2$

Marvin Kopp,[1,*] Charu Garg,[1] Sarah Krebber,[1] Kristin Kliemt,[1] Cornelius Krellner,[1] Sudhaman R. Balguri,[2] Mira Mahendru,[2] Fazel Tafti,[2] Theodore L. Breeze,[3] Nathan P. Bentley,[3] Francis L. Pratt,[4] Thomas J. Hicken,[5] Hubertus Luetkens,[5] Jonas A. Krieger,[5] Stephen J. Blundell,[6] Tom Lancaster,[3] M. Victoria Ale Crivillero,[7] Steffen Wirth,[7] and Jens Müller[1,†]

[1] *Physikalisches Institut, Goethe-University Frankfurt*
Max-von-Laue Str. 1, 60438 Frankfurt am Main, Germany
[2] *Department of Physics, Boston College*
140 Commonwealth Avenue, Chestnut Hill, MA 02467, USA
[3] *Department of Physics, Durham University, Durham, DH1 3LE, United Kingdom*
[4] *ISIS Facility, STFC-Rutherford Appleton Laboratory,*
Harwell Science and Innovation Campus, Didcot, OX11 0QX, United Kingdom
[5] *PSI Center for Neutron and Muon Sciences, 5232 Villigen PSI, Switzerland*
[6] *Department of Physics, Oxford University, Clarendon Laboratory, Parks Road, Oxford, OX1 3PU, UK*
[7] *Max-Planck-Institute for Chemical Physics of Solids*
Nöthnitzer Str. 40, 01187 Dresden, Germany

(Dated: January 9, 2026)

I. SUPPLEMENTARY INFORMATION

A. Sample analysis

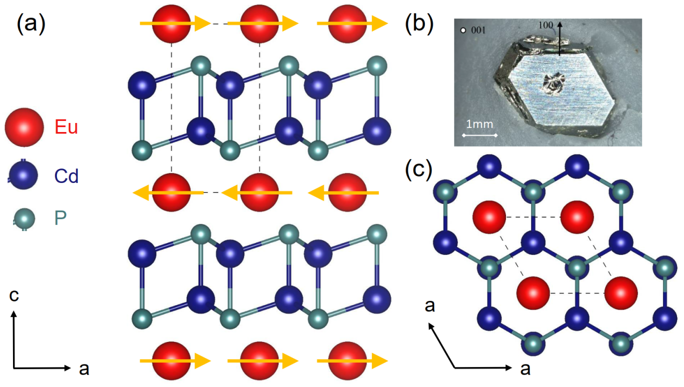

FIG. S1. Structure of $EuCd_2P_2$ in (a) and (c) with A-type AFM order indicated by yellow arrows. (b) shows sample #1 after polishing and before gold evaporation and contacting.

The structure and chemical composition of the $EuCd_2P_2$ samples was confirmed using powder X-ray diffraction (PXRD), energy dispersive X-ray spectroscopy (EDX) and single crystal analysis. The characterisation of the crystal structure of sample #1 by PXRD yielded lattice parameters of $a = 4.324$ Å and $c = 7.179$ Å. The powder diffraction patterns were recorded on a diffractometer with a Bragg-Brentano geometry and copper K_α radiation (Bruker D8).The chemical composition of sample #1 was analysed with EDX, yielding averaged values of Eu= (17 ± 2) at%, Cd= (40 ± 2) at% and P $= (43 \pm 2)$ at%. Analyis of sample #2 yielded similar results within the error range.

In Tab. S1 we provide a comparison of various parameters relevant for the magnetotransport properties for different samples reported in the literature and in this work. Interestingly, samples exhibit both metallic or semiconducting

* kopp@physik.uni-frankfurt.de
† j.mueller@physik.uni-frankfurt.de

behavior upon cooling from room temperature and a significant range of charge carrier concentration n_c. Previous reports have established a correlation between higher charge densities and Eu vacancies, suggesting the possibility of intrinsic doping [3]. Samples reported in [3] are even metals with low $\rho(300\,\mathrm{K})$ and a positive coefficient $\mathrm{d}\rho/\mathrm{d}T > 0$ down to low temperatures where FM ordering occurs, highlighting the strong competition between FM and AFM interactions in this material. The magnitude of the CMR and also the ratio $\rho(T_{\mathrm{peak}})/\rho(300\,\mathrm{K})$ systematically increase with decreasing n_c, wheras T_{peak} increases with increasing n_c.

Sample/Data origin	#1 [this work]	#2 [this work]	Wang *et al.* [1]	Zhang *et al.* [2]	Chen *et al.* [3]
$n_c(300\,\mathrm{K})$ from Hall	$4.8 \cdot 10^{17}\,\mathrm{cm}^{-3}$	$3.5 \cdot 10^{18}\,\mathrm{cm}^{-3}$	$-6 \cdot 10^{18}\,\mathrm{cm}^{-3}$	$\sim 1 \cdot 10^{17}\,\mathrm{cm}^{-3}$	$4.6 \cdot 10^{19}\,\mathrm{cm}^{-3}$ (at 150 K)
$\rho(300\,\mathrm{K})$	$2.3 \cdot 10^{-1}\,\Omega\mathrm{cm}$	$1.4\,\Omega\mathrm{cm}$	$2.4 \cdot 10^{-2}\,\Omega\mathrm{cm}$	$3.1 \cdot 10^{-1}\,\Omega\mathrm{cm}$	$5.5 \cdot 10^{-3}\,\Omega\mathrm{cm}$
high-T behavior	semiconducting	semiconducting	metallic	n.a.	metallic
$\rho(T_{\mathrm{peak}})/\rho(300\,\mathrm{K})$	$1.33 \cdot 10^4$	$1.46 \cdot 10^3$	$5 \cdot 10^1$	$5 \cdot 10^3$	0.87
T_{peak}	14 K	15 K	18 K	14 K	47 K
T_{N}	11 K	11 K	11 K	10.9 K	$T_{\mathrm{C}} = 47\,\mathrm{K}$
$\mathrm{MR}_{\mathrm{max}}$	$2.5 \cdot 10^5\,\%$ (14 K, 5 T)	$3.5 \cdot 10^4\,\%$ (16 K, 5 T)	$5.6 \cdot 10^3\,\%$ (18 K, 5 T)	$1.75 \cdot 10^5\,\%$ (14 K, 2 T)	80% (47 K, 5 T)
$\mu(300\,\mathrm{K}) = (\rho e n_c)^{-1}$	$56\,\mathrm{cm}^2/\mathrm{Vs}$	$1.3\,\mathrm{cm}^2/\mathrm{Vs}$	$-43.3\,\mathrm{cm}^2/\mathrm{Vs}$	$\sim 200\,\mathrm{cm}^2/\mathrm{Vs}$	$24.6\,\mathrm{cm}^2/\mathrm{Vs}$

TABLE S1. Properties of different $EuCd_2P_2$ samples. Zhang *et al.* report sample-to-sample dependencies and some information is not available (n.a.).

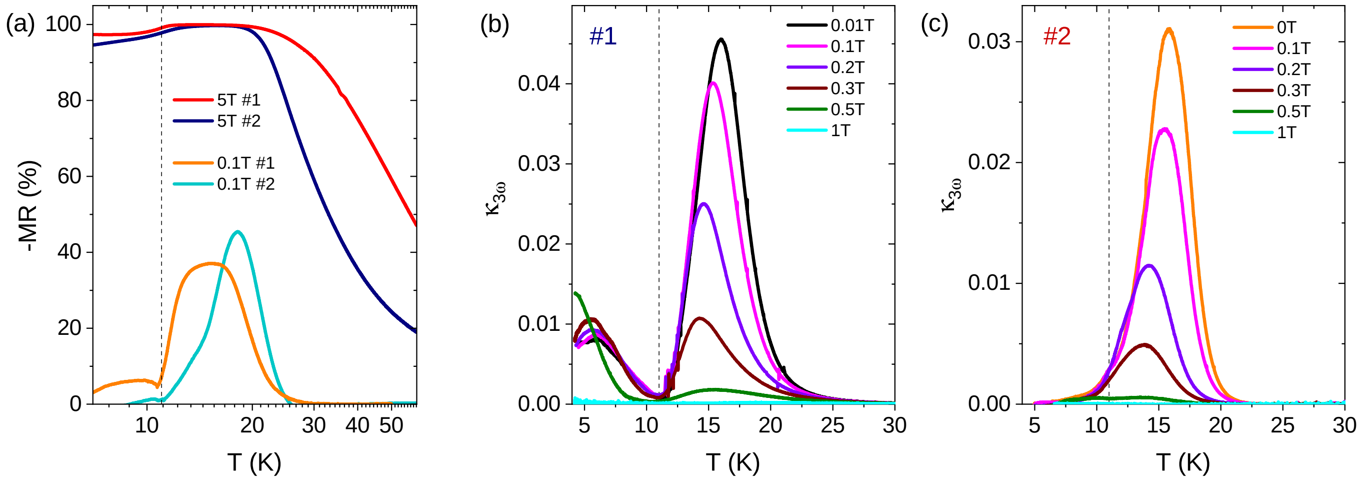

FIG. S2. (a) Negative magnetoresistance $-MR = -(\rho(B) - \rho(0))/\rho(0)$ at relatively small ($\mu_0 H = 0.1\,\mathrm{T}$) and large fields $\mu_0 H = 5\,\mathrm{T}$ for both samples #1 (in orange and red) and #2 (in light blue and blue). (b,c) Fourier coefficient $\kappa_{3\omega} = V_{3\omega}/V_{1\omega}$ of the third-harmonic voltage generation below $T = 30\,\mathrm{K}$ for sample #1 and #2 measured in magnetic fields from $\mu_0 H = 0\,\mathrm{T}$ to $1\,\mathrm{T}$.

B. Weakly-nonlinear resistance and noise

Besides the common resistance calculated by Ohm's law $R = V/I$, assuming a linear dependence between current I flowing through, and voltage drop V across the sample, in spatially inhomogeneous systems it is often useful to consider so-called higher harmonics of the voltage signal. Prime examples for intrinsic electronic and/or magnetic phase separation are the vortex lattice in a type-II superconductor, the magnetic skyrmion lattice, coexistence of phases near a first-order phase transition or the emergence of nano-scale clusters (e.g. polar nanoregions in relaxor ferroelectrics or magnetic polarons in CMR systems) [4–10]. The latter two examples are often discussed in the context of percolation in a random resistor network (RRN) [11, 12]. Basic ideas of such a scenario are often invoked to describe the dynamics of magnetic polarons in CMR systems [13, 14].
In a model percolation system of a semicontinuous metal film [15], upon application of an AC current ($I = I_0 \cos(\omega t)$ the local resistance r_α can be written as

$$r_\alpha = r_0 + \delta r_\alpha \cos(2\omega t + \phi)\,, \tag{1}$$

where δr_α are the fluctuations of each local resistance assumed to be significantly smaller than their resistance r_0 and ϕ is the phase shift between the heat production and the local temperature. The voltage across the sample can be written as

$$V = IR = I_0 R_0 \cos(\omega t) + \frac{1}{2} I_0 \Delta R \cos(3\omega t + \phi)\,. \tag{2}$$

Thus, a third-harmonic voltage

$$V_{3\omega} \propto I_0 \Delta R \propto \frac{\sum i_\alpha^4 r_\alpha^2}{I_0} \tag{3}$$

is created by the sample and connected to the fourth-order distribution of the local currents i_α. In a simple RNN there is a direct connection to the PSD of the resistance fluctuations:

$$S_R/R^2 \propto \frac{V_{3\omega}}{I_0^3 R^2} \tag{4}$$

Here, energy conservation $I^2 \Delta R = \sum i_\alpha^2 \delta r_\alpha$ is used and a power-law scaling

$$\frac{V_{3\omega}}{I_0^3} \propto R^{2+w} \tag{5}$$

is expected, where the critical exponent $w = \kappa/t$ depends on the particular percolation scenario [16, 17].

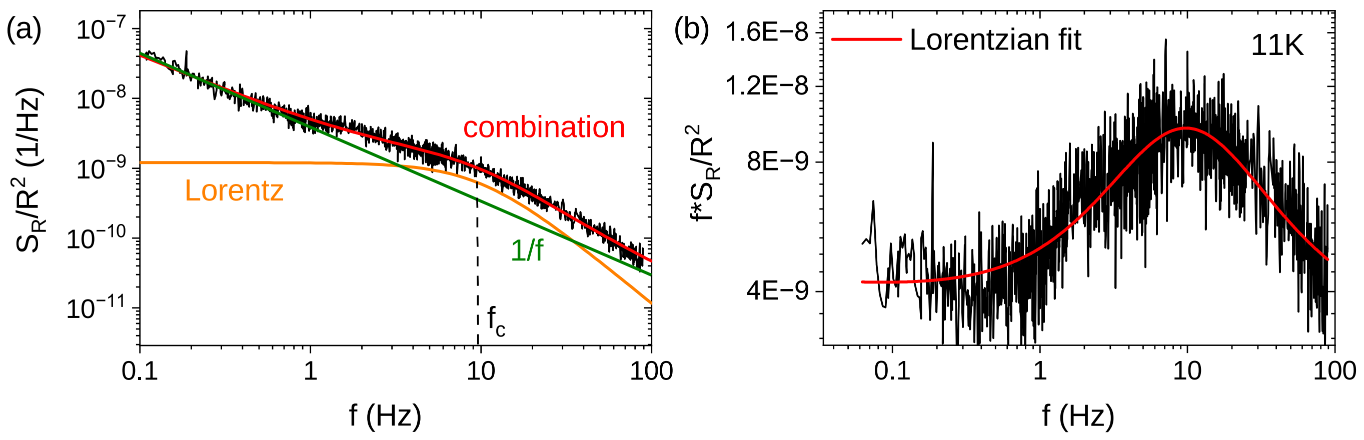

FIG. S3. Typical PSD spectrum $S_R/R^2(f)$ of a $1/f-$type noise (green line) superimposed with a dominating two-level fluctuator producing a Lorentz spectrum (orange) in (a). Plot of $S_R/R^2 \cdot f$ with a fit (red) to evaluate the amplitude of the $1/f$-background, it's frequency exponent α, the amplitude of the lorentzian and the corner frequency f_c (b).

The generated third-harmonic resistance or so-called weakly-nonlinear transport can be easily accessed in an AC lock-in measurement and reveals sensitive information on microscopic inhomogeneities in the current distribution of CMR systems [9, 18]. The results are shown in Fig. S2 for #1 in (b) and for #2 in (c). The Fourier coefficient $\kappa_{3\omega} = V_{3\omega}/V_{1\omega}$ and is clearly nonzero above $T_{\rm N}$ and below about 25 K and shows a pronounced peak for both samples at about ~ 16 K in the vicinity of the resistance peak. We note that the observed values of $\kappa_{3\omega} \sim 3 - 4\,\%$ in zero magnetic field are similarly large as the ones observed for the FM CMR systems EuB_6 (bulk) [9] and (La,Ca)MnO_3 (thin film) [18], where the third-harmonic generation is due to a percolative insulator-metal transition involving magnetic polarons.
Strikingly, the weakly-nonlinear transport peak is strongly suppressed already in small magnetic fields, with the peak shifting to lower temperatures with increasing field, and vanishes between about $\mu_0 H = 0.5$ T and 1 T for both samples. According to the common interpretation outlined above, this means a significant inhomogeneity in the microscopic current distribution in a rather well defined temperature interval above $T_{\rm N}$, which becomes suppressed in moderate magnetic fields. We note that the temperatures where $\kappa_{3\omega} \gtrsim 0$ coincides with a finite negative MR at low fields and with the plateau-like regime of a large negative MR of more than 99 % at higher fields. Again, in agreement with the behavior of the MR at smaller fields, for sample 1, a second, smaller peak develops upon cooling below $T_{\rm N}$, the magnitude of which changes only slightly in increasing magnetic fields and is only suppressed in this temperature regime for fields of order 1 T. Sample 2 does not show such a second peak and the weakly-nonlinear transport is close to zero below $T_{\rm N}$.

An exemplary measured power spectral density (PSD) S_R/R^2 of the resistance fluctuations is shown in Fig. S3 (a) in a double logarithmic plot, where the Lorentzian contribution is shown in blue, the linear $1/f$ background in red and the resulting combination in orange, describing the measured spectrum in black. Also marked is the corner frequency f_c of the Lorentzian spectrum, which is connected to the lifetimes of the two-level fluctuator by $f_c = 1/(2\pi) \cdot (1/\tau_1 = 1/\tau_2)$. By plotting $S_R/R^2 \cdot f$ as shown in (b), one can fit the spectrum with a Lorentzian function and obtain the amplitude and slope of the $1/f$ contribution, as well as the corner frequency f_c and the amplitude of the lorentzian contribution.

In a percolation scenario a power-law dependence $B = V_{3\omega}/I^3 \propto R^{2+w}$ should be observed for the weakly nonlinear resistance [15]. As shown in Fig. S4 the data can be linearly fitted between $T = 18$ K and $T = 15$ K yielding $w = 1.24$ very similar to the observation in silver thin films (or $w = 0.53$ for $T < 15\,K$). A fit in the same temperature range for $S_R/R^2(f = 1\,\text{Hz})$ plotted versus $R(T)$ yields a larger $w = 4.4$ but also with a much larger error ± 0.7 as the fit is worse and there are less points to average over (or $w = 1.18 \pm 0.47$ below $T = 15\,K$). In theory the scaling should also be $S_R/R^2 \propto R^w$. It should be mentioned, that scaling of the third-harmonic signal with current and frequency, as well as basic scaling relations of the noise PSD expected for simple RRNs do not yield the same results for the present system, but vary with the chosen temperature range, which likely is due to the intricate interplay between competing AFM and FM interactions in $EuCd_2P_2$ (see Fig. S4).

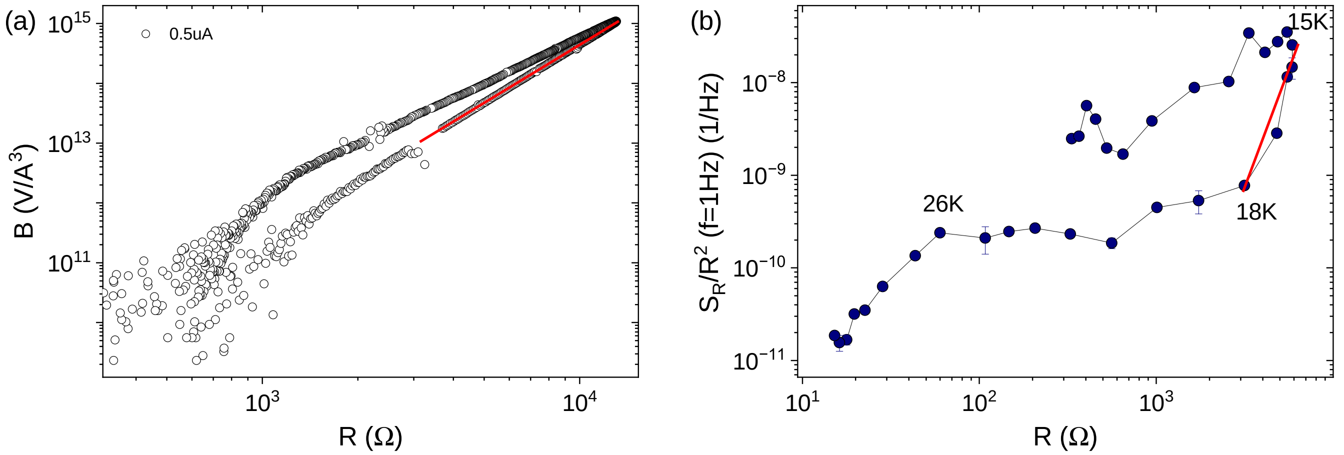

FIG. S4. Plot of the normalized third-harmonic amplitude $B(T) = V_{3\omega}(T)/I^3$ versus the linear resistance $R(T)$ on a double logarithmic scale (a). The data can be linearly fitted between $T = 18\,\mathrm{K}$ and $T = 15\,\mathrm{K}$ yielding $w = 1.24$. A fit in the same temperature range for $S_R/R^2(f = 1\,\mathrm{Hz})$ plotted versus $R(T)$ (b) yields a larger $w = 4.4$ but also with a much larger error ± 0.7.

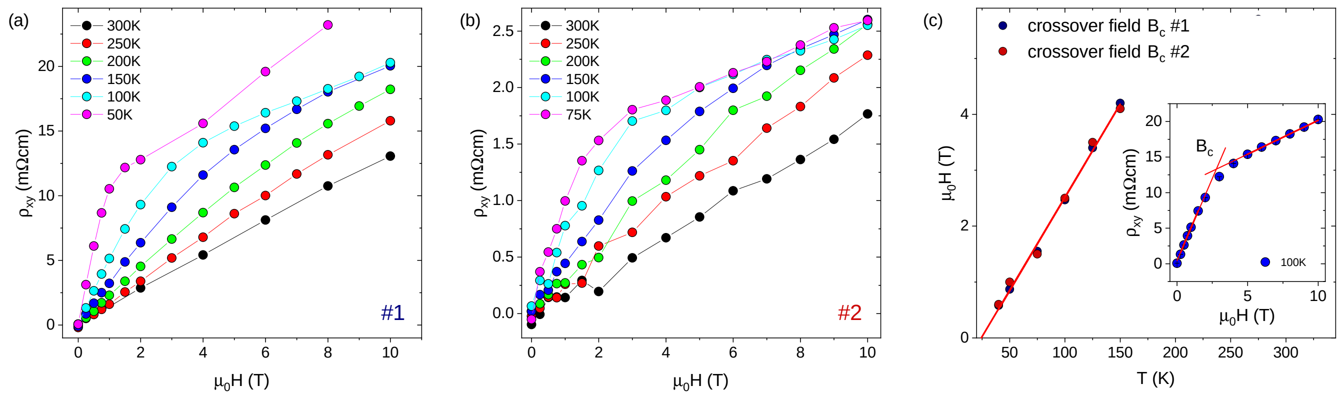

FIG. S5. (a) Hall resistivity ρ_{xy} measured at distinct temperatures are shown for sample #1 and #2 in (b). The linear behavior at high temperatures develops into a curved shape upon cooling with two distinct slopes at low and high magnetic fields. Lines are guides to the eyes. (c) Crossover field B_c where the two slopes intersect, shown for both samples #1 and #2 in blue and red, respectively. Inset shows the construction at $T = 100\,\mathrm{K}$.

C. Magnetic and magnetotransport measurements

Hall measurements were carried out on the same samples #1 and #2 using the given contact geometry. In order to cancel out the magnetoresistance offset due to the non-perfect Hall cross geometry, voltages have been measured at discrete positive and negative magnetic fields antisymmetrized.

Figure S5(a) displays the Hall resistivity ρ_{xy} vs. $\mu_0 H$ up to 10 T for various discrete temperatures from $T = 300\,\mathrm{K}$ down to 50 K for sample %1, see SI Fig. S5 for the data of #2. The temperature profiles for both samples are rather similar, despite the about one order of magnitude larger slope at room temperature (and correspondingly lower carrier concentration) of #1, see inset of Fig. 1(b). In particular, the almost perfect scaling of the crossover field B_c for both samples shown in Fig. S5(c), together with the power-law scaling of the weakly-nonlinear transport as evidence for dynamic percolation, Fig. S4(a), and the strong magnetic field dependence of the electrical transport, rules out extrinsic effects like structural/chemical inhomogeneities.

In order to compare low-frequency resistance and magnetic fluctuations we measured the frequency-dependent AC susceptibility of sample #2. Figure S6(a) shows the real part $\chi'(T,f)$ measured at zero external DC field with an AC field of 5 Oe applied along the c-axis for different frequencies (yellow to red colors) in comparison to the data taken at $\mu_0 H = 5\,\mathrm{T}$ dc field (blue) for $f = 17\,\mathrm{Hz}$. For decreasing temperature the susceptibility shows the expected behavior for an antiferromagnet with an essentially frequency-independent cusp marking the magnetic transition temperature $T_\mathrm{N} = 11\,\mathrm{K}$ and further decrease in the AFM phase. The jump at $T = 3.7\,\mathrm{K}$ is due to the superconducting transition of Sn flux incorporated in to the sample during the crystal growth. In a magnetic field of 5 T the susceptibility decreases below about $T = 40\,\mathrm{K}$ reaching zero below $T = 17\,\mathrm{K}$.

The imaginary part of the AC susceptibility $\chi''(T)$ is shown in Fig. S6(b) for frequencies $f = 17 - 477\,\mathrm{Hz}$. The magnitude of $\chi''(T)$ with a sharp peak at $T_\mathrm{N} = 11.1\,\mathrm{K}$ is frequency dependent with larger values for higher frequencies. (The anomaly at $T = 3.7\,\mathrm{K}$ is due to the superconducting transition of Sn flux incorporated in the sample during crystal growth.)

In the inset of Fig. S6(a) the anisotropy $\gamma(T) = \chi'_{001}(T)/\chi'_{210}(T)$ for the AC field along the c-axis (hard axis) and within the a-a plane (easy axis) is shown. We find $\gamma(T) \approx 1$ (dotted line) at high temperatures while it starts to gradually decrease upon cooling until a sharp downturn occurs below $T \approx 25\,\mathrm{K}$, i.e. the susceptibility for the field applied in the a-a plane is increasing faster and becomes larger than for the c-direction. Below the magnetic ordering temperature, the anisotropy in the zero-field magnetic susceptibility saturates at a value of $\gamma \sim 0.14$.

In the inset of Fig. S6(c) the calculated $S_M(f)$ at $T = 11.2\,\mathrm{K}$ is shown to follow a $1/f^\alpha$ frequency dependence with $\alpha = 0.98$, allowing a direct comparison to the resistance noise measurements which we present in the discussion chapter.

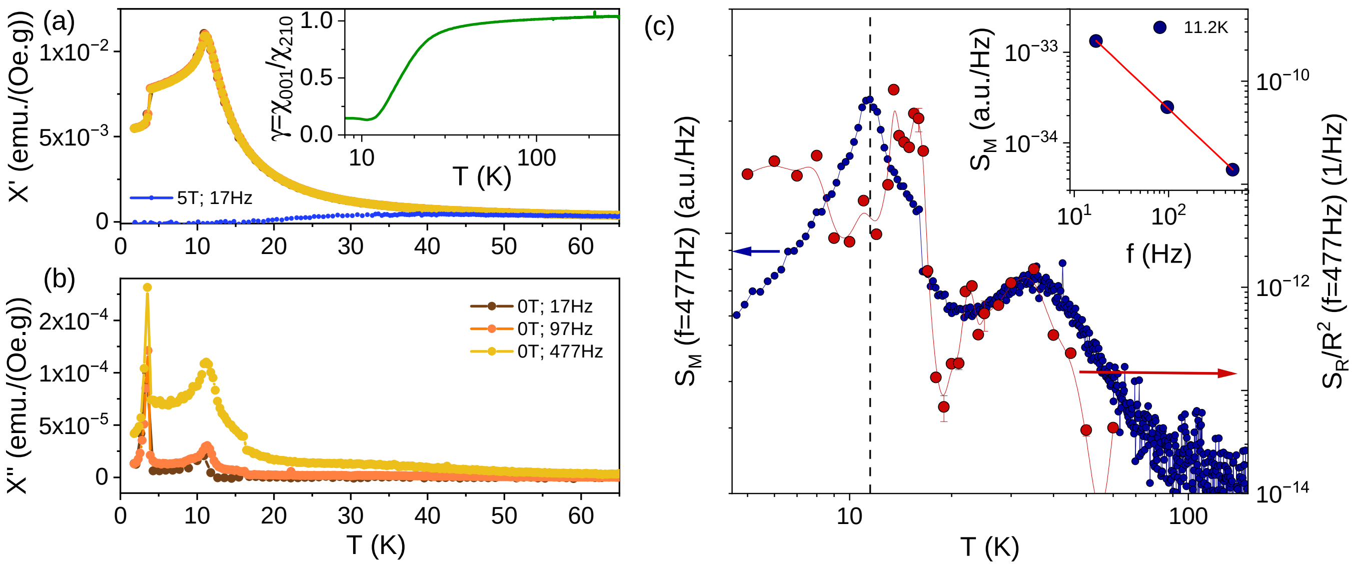

FIG. S6. (a) Real part of the AC susceptibility χ' of sample #2 at zero dc magnetic field for various frequencies between $f = 0.97\,\mathrm{Hz}$ and $f = 477\,\mathrm{Hz}$. Data at 17 Hz measured in an external dc field of $\mu_0 H = 5\,\mathrm{T}$ are shown in blue. The amplitude of the AC field is $\mu_0 H = 0.5\,\mathrm{mT}$. Inset shows the anisotropy $\gamma(T) = \chi'_{001}(T)/\chi'_{210}(T)$, i.e. the ratio of the zero dc field susceptibilities with the AC field along the c-axis and within the a-a plane. (b) Imaginary part χ'' with the same colour code for different frequencies. (c) Comparison of the calculated magnetic noise PSD $S_M(f = 477\,\mathrm{Hz})$ (blue) and the measured resistance noise PSD $S_R/R^2(f = 477\,\mathrm{Hz})$ (red). Inset demonstrates the $1/f$ dependence of the magnetic noise at $T = 11.2\,\mathrm{K}$.

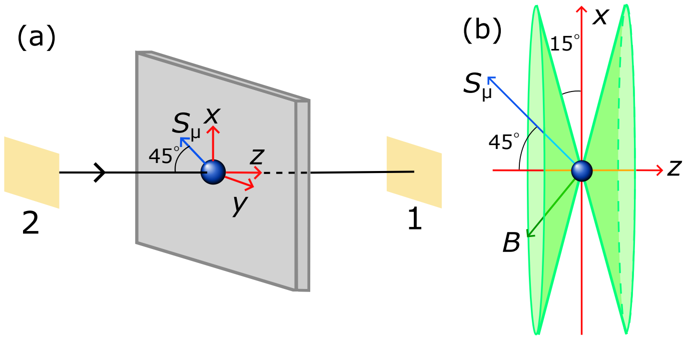

FIG. S7. The geometry of our μSR experiments made at SμS. (a) The detector setup, showing the initial muon spin orientation relative to the beam direction and the forwards (1) and backwards (2) detectors. (b) The cone of possible local dipole fields, B, at the muon stopping site, resulting in a range of possible angles, ϕ, between the muon spin and the local field.

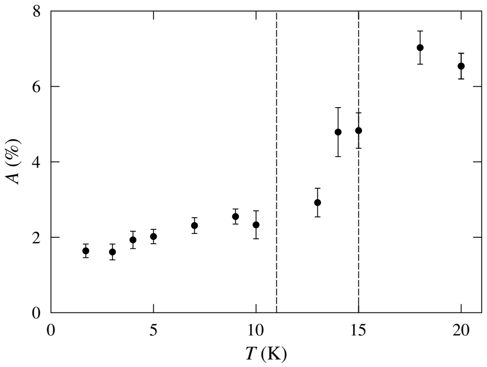

FIG. S8. (a) The amplitude, A, of the asymmetry component in our wTF experiments at SμS corresponding to oscillations in an applied field.

D. Muon-spin relaxation

The ZF μSR data measured at SμS is made up of two components at temperatures below T_{N}: an oscillating term with an asymmetry of $A = 9.0\,\%$ and a constant term with $A = 4.7\,\%$. We made weak transverse field (wTF) measurements at SμS where a field of 3 mT is directed along the x-direction in Fig. S7(a), which allow us to observe the response of muon spins in magnetically disordered environments. The amplitude of the nonmagnetic asymmetry component is plotted against temperature in Fig. S8 and shows an amplitude of 1.5 % at the lowest temperature point. This contribution could be either a background due to muons stopping outside the sample or a behavior intrinsic to the material, implying the existence of a small nonmagnetic volume fraction at low temperatures. In the absence of any other evidence for the latter case, we assume that this term represents a 1.5 % background in all of our data collected at SμS. The amplitude of the constant term in the ZF data with this background subtracted is therefore 3.2 %. The oscillating term corresponds to the precession of the component of the muon spin that is perpendicular to the local field, and therefore, in a single crystal sample, has an amplitude proportional to the sine squared of the spin-field angle, ϕ. The constant term corresponds to the component of the muon spin that is pinned parallel to the local field, and so has an amplitude proportional to $\cos^2\phi$. The ratio of the two is therefore $\tan^2\phi = 9.0/3.2 = 2.8 \pm 0.99$ or $\phi = 59 \pm 5°$.

As part of our analysis of the μSR data we carried out DFT calculations to identify a candidate muon stopping site at a single low energy position within the unit cell (as discussed in Section IV D). We were able to then calculate the local dipole field experienced by muons stopping at this site, which is found to be pointed 15° out of the $a-a$ plane. In our experiments the spins of the incoming muons were polarized at an angle of 45° to their direction of travel towards the sample (the z axis), as shown in Fig. S7(a). The sample was positioned such that the crystallographic c axis was orientated along z, so that the local dipole field at the muon site must exist on a cone about the z axis depending on the crystal orientation in the $a - a$ plane as shown in Fig. S7(b). The minimum spin-field angle is therefore $\phi = 30°$, so that $0.33 \leq \tan^2\phi \leq \infty$, compatible with our estimate of $\phi = 59°$ from the data. This is complicated slightly by the fact that in our experiments we used a mosaic made up of single crystals oriented in this way. The resulting

averaging over angles in the $a-a$ plane gradually constrains this range with increasing crystal number, approaching an expected value of 1.14 [19], but for a small number our observed ratio of 2.8 is reasonable.

Over the temperature region $T_{\rm N} < T < T^*$ the total asymmetry rises from 13.7 % to 16.6 %. Accounting for a background of 1.5 %, this gives sample-only asymmetries of 12.2 % and 15.1 % respectively. The 45° rotation of the initial muon spin away from the detector axis means that the total initial asymmetry is reduced by a factor of $\sqrt{2}$, so at high temperatures $16.6\% \times \sqrt{2} = 23.5\,\%$ is in good agreement with the expected total asymmetry on the FLAME instrument. This suggests that we are observing the full asymmetry from all stopped muons in the high-temperature region, but that at low temperatures there is a phase accounting for 0.19 ± 0.02 of the total sample volume whose contribution is not observed in the asymmetry, presumably because the spins in this magnetic environment are relaxed so quickly that they are not resolved in our data.

Our wTF measurements show an increase of about 4.5 % above $T_{\rm N}$. This corresponds to a total volume fraction of 0.30 ± 0.06 undergoing a phase transition from magnetic order to nonmagnetic disorder. The rest of the muon sites from the ordered phase therefore enter a dynamically fluctuating magnetic phase that strongly relaxes the muon spins.

---

[1] Z. Wang, J. D. Rogers, X. Yao, R. Nichols, K. Atay, B. Xu, J. Franklin, I. Sochnikov, P. J. Ryan, D. Haskel, and F. Tafti, Colossal Magnetoresistance without Mixed Valence in a Layered Phosphide Crystal, Advanced Materials **33**, 2005755 (2021).

[2] H. Zhang, F. Du, X. Zheng, S. Luo, Y. Wu, H. Zheng, S. Cui, Z. Sun, Z. Liu, D. Shen, M. Smidman, Y. Song, M.Shi, Z. Zhong, C. Cao, H. Yuan, and Y. Liu, Electronic band reconstruction across the insulator-metal transition in colossally magnetoresistive $EuCd_2P_2$, Phys. Rev. B **108**, L241115 (2023).

[3] X. Chen, Z. Wang, Z. Zhou, W. Yang, Y. Liu, J.-Y. Lu, Z. Ren, G.-H. Cao, F. Tafti, S. Dong, and Z.-C. Wang, Manipulating magnetism and transport properties of $EuCd_2P_2$ with a low carrier concentration, Physical Review B **109** (2024).

[4] G. Blatter, M. V. Feigel'man, V. B. Geshkenbein, A. I. Larkin, and V. M. Vinokur, Vortices in high-temperature superconductors, Rev. Mod. Phys. **66**, 1125 (1994).

[5] N. Nagaosa and Y. Tokura, Topological properties and dynamics of magnetic skyrmions, Nature Nanotechnology **8**, 899 (2013).

[6] T. Thomas, Y. Agarmani, S. Hartmann, M. Kartsovnik, N. Kushch, S. M. Winter, S. Schmid, P. Lunkenheimer, M. Lang, and J. Müller, Slow and non-equilibrium dynamics due to electronic ferroelectricity in a strongly-correlated molecular conductor, npj Spintronics **2** (2024).

[7] P.-G. de Gennes, Effects of Double Exchange in Magnetic Crystals, Physical Review **118**, 141 (1960).

[8] T. Kasuya and A. Yanase, Anomalous Transport Phenomena in Eu-Chalcogenide Alloys, Reviews of Modern Physics **40**, 684 (1968).

[9] P. Das, A. Amyan, J. Brandenburg, J. Müller, P. Xiong, S. von Molnár, and Z. Fisk, Magnetically driven electronic phase separation in the semimetallic ferromagnet EuB_6, Phys. Rev. B **86**, 184425 (2012).

[10] M. Pohlit, S. Rößler, Y. Ohno, H. Ohno, S. von Molnár, Z. Fisk, J. Müller, and S. Wirth, Evidence for Ferromagnetic Clusters in the Colossal-Magnetoresistance Material EuB_6, Physical Review Letters **120**, 10.1103/physrevlett.120.257201 (2018).

[11] R. Rammal, C. Tannous, P. Breton, and A.-M. S. Tremblay, Flicker ($1/f$) Noise in Percolation Networks: A New Hierarchy of Exponents, Phys. Rev. Lett. **54**, 1718 (1985).

[12] D. Stauffer and A. Aharony, *Introduction To Percolation Theory* (Taylor & Francis, 2018).

[13] L. P. Gor'kov and V. Z. Kresin, Properties of Manganites: Band and Percolation Approaches, Journal of Superconductivity **12**, 243 (1999).

[14] A. Kaminski and S. D. Sarma, Polaron Percolation in Diluted Magnetic Semiconductors, Physical Review Letters **88**, 10.1103/physrevlett.88.247202 (2002).

[15] Y. Yagil and G. Deutscher, Third-harmonic generation in semicontinuous metal films, Phys. Rev. B **46**, 16115 (1992).

[16] A.-M. S. Tremblay, S. Feng, and P. Breton, Exponents for $1/f$ noise, near a continuum percolation threshold, Physical Review B **33**, 2077 (1986).

[17] S. Kogan, *Electronic noise and fluctuations in solids* (Cambridge University Press, 1996).

[18] V. Moshnyaga, K. Gehrke, O. I. Lebedev, L. Sudheendra, A. Belenchuk, S. Raabe, O. Shapoval, J. Verbeeck, G. V. Tendeloo, and K. Samwer, Electrical nonlinearity in colossal magnetoresistance manganite films: Relevance of correlated polarons, Phys. Rev. B **79**, 134413 (2009).

[19] J. Graham, T. Hicken, R. Regmi, M. Janoschek, I. Mazin, H. Luetkens, N. Ghimire, and Z. Guguchia, Local probe evidence supporting altermagnetism in $Co_{1/4}NbSe_2$, arXiv preprint arXiv:2503.09193 (2025).
